# SARS-CoV-2 IgG seroprevalence in blood donors located in three different federal states, Germany, March to June 2020

**DOI:** 10.2807/1560-7917.ES.2020.25.28.2001285

**Published:** 2020-07-16

**Authors:** Bastian Fischer, Cornelius Knabbe, Tanja Vollmer

**Affiliations:** 1 Herz- und Diabeteszentrum NRW, Institut für Laboratoriums- und Transfusionsmedizin, Bad Oeynhausen, Germany

**Keywords:** SARS-CoV-2, COVID-19, anti-SARS-CoV-2 IgG seroprevalence in Germany

## Abstract

Most cases of coronavirus disease 2019 are mild or asymptomatic. Therefore, many cases remain unrecorded. We determined seroprevalence of IgG antibodies against severe acute respiratory syndrome coronavirus 2 (SARS-CoV-2) in 3,186 regular blood donors in three German federal states between 9 March and 3 June 2020. The IgG seroprevalence was 0.91% (95% confidence interval (CI): 0.58–1.24) overall, ranging from 0.66% (95% CI: 0.13–1.19) in Hesse to 1.22% (95% CI: 0.33–2.10) in Lower-Saxony.

Common symptoms of coronavirus disease 2019 (COVID-19) include cough, fever and respiratory problems. While ca 80% of infected people only show mild or no symptoms, some develop severe pneumonia, multiple organ failure or even die [1]. Current estimates assume a mortality rate of ca 2% in medically attended patients [2]. However, individuals with mild or no symptoms are not all included in these mortality estimates, and the number of unrecorded cases is unknown [3,4]. Although an acute infection with severe acute respiratory syndrome coronavirus 2 (SARS-CoV-2) is usually verified by PCR, a recent publication suggests a positive identification of anti-SARS-CoV-2 IgG antibodies as an acceptable approach to confirm infection [5]. 

To determine an approximation of the actual rate of people who have recovered from COVID-19, representative of the German population, we determined the anti-SARS-CoV-2 IgG seroprevalence of regular blood donors resident in three different German federal states between March and June 2020.

## Presence of anti-SARS-CoV-2 IgG in blood donors

Residual material leftover from routine diagnostics from 3,186 regular blood donors without any preselection (2,257 (70.84%) men and 929 (29.16%) women), donated in the period between 9 March and 3 June 2020, were screened for the presence of anti-SARS-CoV-2 IgG directed against domain S1 of the SARS-CoV-2 spike protein using the anti-SARS-CoV-2 enzyme-linked immunosorbent assay (ELISA) from Euroimmun (Lübeck, Germany). In recent publications, this serological ELISA showed a high specificity of 99–100% and a sensitivity of ca 65% [6-9]. Semiquantitative results were calculated as the ratio of the extinction of samples over the extinction of a calibrator. Seropositive results were confirmed using the Architect SARS-CoV-2 IgG (Abbott, Wiesbaden, Germany) targeting the viral nucleocapsid and the LIAISON SARS-CoV-2 S1/S2 IgG assay (DiaSorin Deutschland GmbH, Dietzenbach, Germany) targeting the SARS-CoV-2 spike protein. 

Most samples (2,902/3,186; > 91%) were obtained between 23 March and 22 May 2020. Samples were obtained from donors located in the three German federal states North Rhine-Westphalia (n = 1,700), Lower Saxony (n = 576) and Hesse (n = 910). Measurements were fully automated and processed according to the manufactures protocol using the Euroimmun Analyzer I system. Overall, we found an anti-SARS-CoV-2 IgG seroprevalence of 0.91% (29/3,186; 95% CI: 0.58–1.24) in our cohort; 24 male and five female donors. No statistical difference in seroprevalence was observed between men and women (p = 0.156). Likewise, the seroprevalence did not differ statistically between the three federal states (p = 0.536), but incidence was highest in Lower Saxony (1.22%; 7/576; 95% CI: 0.33–2.10), followed by North Rhine-Westphalia (0.94%; 16/1,700; 95% CI: 0.49–1.39) and Hesse (0.66%; 6/910; 95% CI: 0.13–1.19) (Table).

**Table:** Anti-SARS-CoV-2 IgG seroprevalence in regular blood donors, by region, Germany, March–June 2020 (n = 3,186)

	IgG-positive			IgG-negative	
	n	%	95% CI	n	%
**Overall**	**29**	**0.91 **	**0.58–1.24**	**3,157**	**99.09**
North Rhine-Westphalia (n = 1,700)	16	0.94	0.49–1.39	1,684	99.06
Lower Saxony (n = 576)	7	1.22	0.33–2.10	569	98.78
Hesse (n = 910)	6	0.66	0.13–1.19	904	99.34

CI: confidence interval; SARS-CoV-2: severe acute respiratory syndrome coronavirus 2.

All donors underwent a medical examination before donation, reported that they did not have current or recent diseases and had no physically detectable symptoms of infection such as fever or an increased leukocyte count. None of the seropositive blood donors reported a known positive medical history of SARS-CoV-2 infection. A second retrospective survey for SARS-CoV-2 related symptoms was not conducted.

## Anti-SARS-CoV-2 IgG ratio distribution of seropositive blood donors

The Figure shows the anti-SARS-CoV-2 IgG distribution in blood donors with equivocal (ratio: ≥ 0.8 to < 1.1) and clearly seropositive (ratio: ≥ 1.1) test results. For clarity, values are presented in a histogram, choosing a bin-width of 0.2 (e.g. ratio 1.1–1.3). The 29 seropositive donors showed a broad spectrum of IgG ratios ranging between 1.13 and 8.9. In addition, we identified nine blood donors with equivocal seropositive IgG antibody ratios ranking between 0.8 and 1.08 who were not considered for the seroprevalence calculation.

**Figure:**
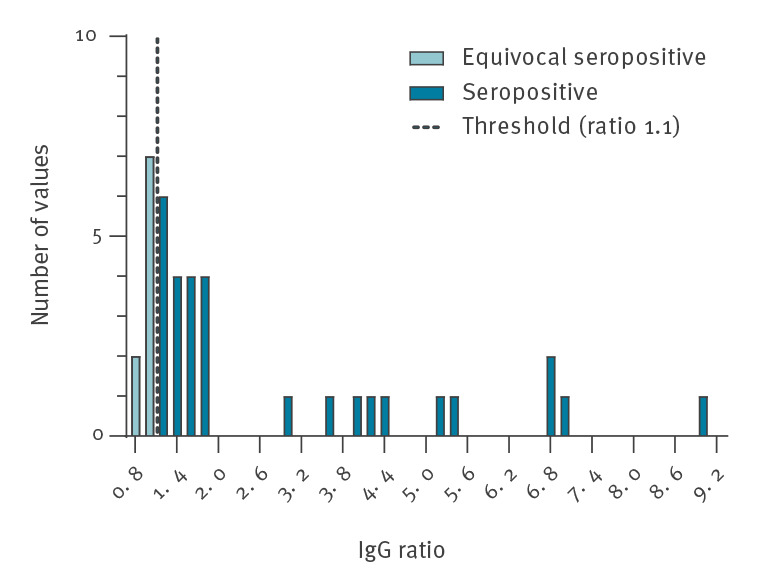
Distribution of anti-SARS-CoV-2 IgG ratios of blood donors with seropositive and equivocal test results, Germany, March–June 2020, (n = 3,186)

SARS-CoV-2: severe acute respiratory syndrome coronavirus 2.

Equivocal were not considered for seroprevalence calculation. The dashed vertical line indicates the IgG seropositivity threshold (ratio ≥ 1.1).

## Ethical statement

We used exclusively waste material from routine laboratory diagnostics, therefore the need for informed consent and ethical approval was waived. Samples were collected in accordance with the German Act on Medical Devices for the collection of human residual material.

## Discussion

After a public festival in February 2020, a local COVID-19 outbreak occurred in Heinsberg, Germany. According to estimations by Streeck et al., this led to an infection rate of 15.5% (positive swab anamnesis and/or detection of anti-SARS-CoV-2 IgG antibodies), whereby 22.2% of infected individuals were asymptomatic [10]. The seroprevalence in non-hotspot regions is currently open to question. In our study, we decided to measure overall anti-SARS-CoV-2 IgG levels instead of neutralising antibodies, as not all individuals who have recovered from COVID-19 seem to express detectable neutralising antibody levels [11]. This would potentially lead to a number of false-negative results. As false-positive measurements could account for a considerable number in populations with a low seroprevalence, initial seropositive measurements were verified with two additional assays (Supplementary Figure S1). Consequently, we revised one initially seropositive tested result. We determined an overall low IgG seroprevalence of 0.91% (95% CI: 0.58–1.24) against SARS-CoV-2 in three German federal states. A recent study of the university hospital Eppendorf revealed comparable values for Hamburg. Their data show that fewer than 1% of 914 tested regular blood donors expressed IgG antibodies against the virus [12]. In addition, a national multicentre study, including four University children`s hospitals in Baden-Württemberg, revealed that 1.3 % of 4,932 individuals (children and their parents) tested between 22 April and 15 May expressed anti-SARS-CoV-2 IgG antibodies [13].

It should be emphasised that the preselection of blood donors as study cohort is accompanied by limitations regarding representation of the population: Blood donors are between 18 and 65 years-old, young healthy adults are usually overrepresented and other groups (e.g. children, HIV/HCV/HBV-infected patients, older people with underlying conditions, institutionalised people) are excluded or underrepresented. Nevertheless, according to official data, only ca 0.2% of German citizens have so far (17 June 2020) been infected with the SARS-CoV-2 [14]. Therefore, our findings suggest that there are a large number of unrecorded cases.

Compared with hotspot regions either in Germany [10] or elsewhere in Europe (e.g. Lombardy, Italy, with a seroprevalence of 23% [15] and Madrid, Spain, with a seroprevalence of 11.5% (95% CI: 9.9–13.3) [16]), the low seroprevalence for SARS-CoV-2 determined in our study could be explained by the imposition of preventive, non-pharmaceutical, interventions at an early stage of the epidemic. A study by Flaxman et al. indicates that these lockdowns contributed considerably to the containment of virus spreading and therefore may have saved many lives [17]. Their model calculation estimating the current SARS-CoV-2 seroprevalence for different European countries revealed the lowest prevalence for Germany (0.85%) and Norway (0.46%), while higher values were estimated for Belgium (8%) and Spain (5.5%). All these rates are far too low to reach herd immunity, which would require ca 60% of the population to express protective antibodies against the virus [18]. 

In addition to the unambiguously seropositive blood donors, 0.3% of the individuals in our cohort showed equivocal levels of antibody. These donors may have been very recently infected and would subsequently have reached higher IgG antibody levels against SARS-CoV-2. However, this assumes that they had an asymptomatic SARS-CoV-2 infection since blood donors represent a selection of apparently healthy individuals lacking any physically detectable symptoms. A longitudinal study on the antibody profile of SARS-CoV-1 patients in 2006 showed that infected persons did not produce detectable antibody titres within the first 7 days after the onset of symptoms; IgG expression increased considerably on day 15 and reached a peak on day 60 [19]. First data for SARS-CoV-2 suggest a very similar timing of IgG antibody formation [20]. In addition, scientists from the university hospital Zurich showed that serum IgG levels remained partially negative in COVID-19 patients with a mild disease progression, whereas severe cases, independently of age, had significantly increased serum IgG titres [21]. Long et al. monitored 285 recovered COVID-19 patients who tested positive for anti-SARS-CoV-2 IgG antibodies within 19 days after symptom onset but also here, seroconversion was delayed in patients with milder symptoms [22]. It is also conceivable, that individuals with a weak humoral immune response (low antibody ratio) have a stronger cellular immune response. In this context, Wu et al. recently presented a negative correlation between lymphocyte counts and neutralising antibody responses to SARS-CoV-2 in a cohort of COVID-19 recovered patients [11]. Interestingly, they also showed that 10 of 175 patients did not express detectable neutralising antibody titres at all. 

## Conclusion

Broad and comprehensive testing is required to better evaluate the number of people who have recovered from COVID-19 and to elucidate the magnitude of unrecorded cases. It has to be taken into consideration that not all convalescents seem to express detectable levels of anti-SARS-CoV-2 IgG antibodies and that there is missing evidence on antibody persistence.

## Supplementary Data

221000 Supplement
