# Supplementary material for: SARS-CoV-2 IgG seroprevalence in blood donors located in three different federal states, Germany, March to June 2020
Source: Euro Surveill. 2020 Jul 16;25(28):2001285. doi: 10.2807/1560-7917.ES.2020.25.28.2001285 (PMC7376847; doi:10.2807/1560-7917.ES.2020.25.28.2001285)
Supplement: Supplement [file 2001285_FISHER_Supplement.pdf]

Supplement

This supplementary material is hosted by *Eurosurveillance* as supporting information alongside the article "**SARS-CoV-2 IgG seroprevalence in blood donors located in three different federal states, Germany, March to June 2020"** on behalf of the authors, who remain responsible for the accuracy and appropriateness of the content. The same standards for ethics, copyright, attributions and permissions as for the article apply. Supplements are not edited by *Eurosurveillance* and the journal is not responsible for the maintenance of any links or email addresses provided therein.

Figure S1: Confirmation of anti-SARS-CoV-2 positive individuals by three different assays.

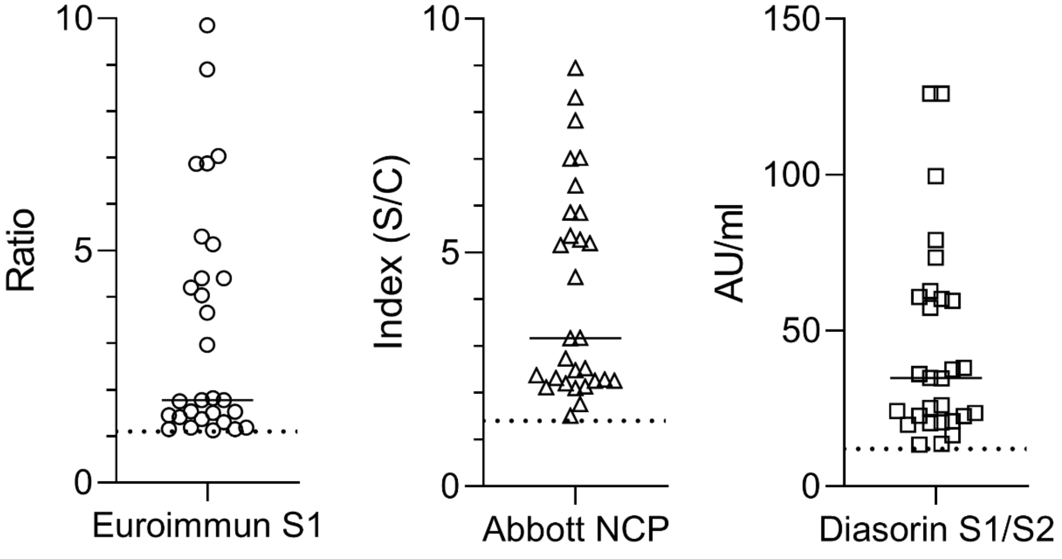

The results of the respective assay were interpreted according to the manufacturer's instruction. The solid horizontal line displays the respective median and the dashed horizontal line displays the cut-off values for positivity of the respective assays (Euroimmun anti-SARS-CoV-2 S1 IgG: $\geq$ 1.1, Abbott Architect SARS-CoV-2 NCP IgG $\geq$ 1.4, Diasorin LIAISON SARS-CoV-2 S1/S2 IgG: $\geq$ 15).
